# Evidence of high EEHV antibody seroprevalence and spatial variation among captive Asian elephants (*Elephas maximus*) in Thailand

**DOI:** 10.1186/s12985-019-1142-8

**Published:** 2019-03-13

**Authors:** Taweepoke Angkawanish, Mirjam Nielen, Hans Vernooij, Janine L. Brown, Peter J. S. van Kooten, Petra B. van den Doel, Willem Schaftenaar, Kannika Na Lampang, Victor P. M. G. Rutten

**Affiliations:** 1 Department of Infectious Diseases and Immunology, Faculty of Veterinary Medicine, Utrecht University, Yalelaan 1, 3584 CL Utrecht, The Netherlands; 2 National Elephant Institute, Lampang-Chiangmai highway (km 28-29), Hangchart, Lampang, 52190 Thailand; 3 Department of Farm Animal Health, Faculty of Veterinary Medicine, Utrecht University, Utrecht, The Netherlands; 4 Center for Species Survival, Smithsonian Conservation Biology Institute, Front Royal, Virginia, USA; 5 ViroScience Lab, Erasmus Medical Center, Rotterdam, The Netherlands; 6 Veterinary Services, Rotterdam Zoo, Rotterdam, The Netherlands; 7 Department of Veterinary Bioscience and Veterinary Public Health, Faculty of Veterinary Medicine, Chiang Mai University, Chiang Mai, Thailand; 8 Department of Veterinary Tropical Diseases, Faculty of Veterinary Science, University of Pretoria, Pretoria, South Africa

**Keywords:** Elephant endotheliotropic herpesvirus, EEHV, Asian elephant, Glycoprotein B ELISA, Seroprevalence, Risk factor

## Abstract

**Background:**

Elephant endotheliotropic herpesviruses (EEHV) can cause an acute highly fatal hemorrhagic disease in young Asian elephants (*Elephas maximus*), both ex situ and in situ. Amongst eight EEHV types described so far, type 1 (subtype 1A and 1B) is the predominant disease-associated type. Little is known about routes of infection and pathogenesis of EEHV, and knowledge of disease prevalence, especially in range countries, is limited.

**Methods:**

A large cross-sectional serological survey was conducted in captive elephants (*n* = 994) throughout Thailand using an EEHV-1A glycoprotein B protein antigen specific antibody ELISA.

**Results:**

Antibody seroprevalence was 42.3%, with 420 of 994 elephants testing positive. Associations between seropositivity and potential risk factors for EEHV infection were assessed and included: elephant age, sex, camp cluster size, management type (extensive versus intensive), sampling period (wet vs. dry season) and location of camp (region). Univariable regression analysis identified management system and region as risk factors for the presence of EEHV antibodies in elephants, with region being significant in the final multivariable regression model. Prevalence was highest in the North region of the country (49.4%).

**Conclusions:**

This study produced baseline serological data for captive elephants throughout Thailand, and showed a significant EEHV burden likely to be maintained in the captive population.

**Electronic supplementary material:**

The online version of this article (10.1186/s12985-019-1142-8) contains supplementary material, which is available to authorized users.

## Background

A number of infectious diseases significantly impact elephant population sustainability, particularly in captivity [1]. Of great concern over the past two decades is infection with the elephant endotheliotropic herpesvirus (EEHV), which can cause hemorrhagic disease (HD). First recognized in 1999 [2], eight genetically-distinct subtypes have been identified, at least six of which are associated with high mortality [3]. In Asian elephants, EEHV-1 (subtypes 1A and 1B) is the most common and virulent, while EEHV-3, − 4 and − 5 are infectious, but rarely fatal [4–7]. Hemorrhagic disease primarily affects Asian elephants under 10 years of age, particularly those between 1 and 4 years, as well as African elephants [8]. Onset of EEHV-HD is rapid, often with few early clinical signs, resulting in death within a few hours to days after presentation of the first clinical signs in ~ 80% of cases that present with the disease [9]. Clinical signs are initially nonspecific, but can include lethargy, lameness and colic, later progressing to include swelling of the head and thoracic limbs, oral ulceration, and cyanosis of the tongue as widespread endothelial cell necrosis occurs [10]. Since its discovery, EEHV has been the cause of 60% of deaths of young captive-born Asian elephants in western zoos, affecting almost one in four Asian elephant calves born in zoos globally [9]. EEHV is not only present in ex situ collections, but has been observed in situ in India [11], Thailand [6], Laos [12], Cambodia, Myanmar, Nepal and Sumatra [3]. Overall, more than 100 deaths from EEHV have been confirmed globally [3, 13], with many more cases likely going undiagnosed.

In Thailand, an earlier study sampled pharyngeal lymph nodes and found no evidence of EEHV in 31 Asian elephants based on PCR [14]. More recently, EEHV infection was confirmed in 15 cases using a semi-nested PCR technique, of which 72% was EEHV-1A [15]. Today, EEHV-1A is considered the major threat among young Thai elephants [16], even though not all elephants infected with EEHV develop symptoms. For example, in Thailand, EEHV-1A was detected in 29 healthy Asian elephants from trunk swab samples [17]. Likewise, positive EEHV results have also been reported for healthy elephants in western zoos [18, 19]. Therefore, it is now recommended that all elephant calves between 1 and 8 years be screened weekly using real time quantitative PCR (qPCR) to monitor viral loads and numbers of monocytes and platelets [18–22]. Elephants with a high viral load and low monocyte and/or platelet counts should be treated immediately with antiviral and antibacterial drugs, as well as with supportive agents to maintain circulatory homeostasis, prevent inflammation which can lead to vascular shock (fluid therapy, plasma transfusion, glucocorticosteroids), and manage pain (NSAID’s) [22]. Unfortunately the efficacy of medications is not only inconsistent, but has yet to be clearly documented. A vaccine against EEHV is not available yet. Although routine testing using qPCR is generally done in a handful of western zoos, testing can be problematic in range countries because of high costs, and limited equipment and expertise. Despite progress made in viral screening by PCR, especially for symptomatic animals, little is known about the pathogenesis and transmission of this disease or about numbers of elephants exposed or infected. Thus, the current lack of sero-epidemiological data presents a significant gap in knowledge of the EEHV disease burden and susceptibility in a species of commercial and cultural importance to Thailand.

Determination of EEHV antibody titers to establish seroprevalence rates among various elephant populations is based on detection of antibodies against an EEHV-1A viral envelope protein, glycoprotein B (gB). Screening of Asian elephants in U.S. and European zoos showed that nearly 80% of PCR positive animals were seropositive for this protein [23]. The aim of the present study was to assess EEHV seroprevalence in a large cross-sectional survey of elephants in Thailand using the EEHV-1A gB protein antigen ELISA [23], and to obtain preliminary data on factors potentially associated with infection throughout the captive population in Thailand.

## Materials and methods

### Animals

The serological survey was conducted retrospectively on serum samples collected between January 2010–February 2015, and comprised elephants (*n* = 994) in private, tourist, and logging camps that were included in a health screening program under the Mobile Elephant Clinic Project in Thailand, led by trained veterinarians and staff of the National Elephant Institute (NEI), Lampang, Thailand. Animals tested represented ~ 25% of the total population of captive elephants in the country. Only elephants deemed healthy (asymptomatic) by veterinarians during the routine examinations were included in this study.

### Data and sample collection

Elephant information recorded consisted of owner name, elephant name, sex, age, microchip number, copy of official registration identity card, present address and health information. Study variables are described in Table 1. Male and female elephants were categorized into three age groups: < 11 years; 11–50 years; and > 50 years. Camps were divided into two types of management systems: intensive and extensive, as defined by Mar [24]. With intensive systems, elephants are managed individually or in small groups, are fed entirely by humans through prepared fodder, and are tethered at night. They may participate in work activities, such as trekking, but often in a more urban setting. Extensive management involves more traditional activities, like logging, forest trekking, and bathing, and releasing elephants into the forest by long chains or hobbles at night to forage and potentially interact with other captive conspecifics. Camps were grouped into six geographical regions, and whether they were close to the border with neighbor countries. Camps, within a radius of 2 km, that shared resources like a river, road, land area, or working area during the day were clustered and categorized based on the number of elephants: small (< 10 elephants/cluster), medium (10–50 elephants) and large (> 50 elephants). Over the 5-year survey, elephants were sampled throughout the year, with data grouped according to wet (April–October) and dry (November–March) seasons in Thailand.

**Table 1:** Study population demographics, and potential risk factors in association with EEHV antibody seroprevalence of elephants in Thailand (*n* = 994)

Risk factors	Category	Number (proportion male/female)
Sex	Female	678 (0.68)
	Male	316 (0.32)
Age category	< 11 years	73 (0.45/0.55)
	11–50 years	797 (0.30/0.70)
	> 50 years	124 (0.29/0.71)
Management type (province^1^)	Extensive: (CM, LP, CR, SKT, Tak, CP)	505 (0.34/0.66) (*n* = 286, 125, 8, 16, 68 and 2 respectively)
	Intensive: (AY, NKPT, RBR, PJ, CBR, Trat, BRR, NKSM, SR, SRTN, PNG, Smui, SKL, CPo)	489 (0.29/0.71) (*n* = 76, 27, 36, 1, 166, 41, 1, 3, 56, 15, 63, 2, 1 and 1 respectively)
Region	Central	76 (0.25/0.75)
	East	207 (0.16/0.84)
	North	435 (0.36/0.64)
	Northeast	62 (0.50/0.50)
	South	82 (0.46/0.54)
	West	132 (0.25/0.75)
Camp cluster^2^	Small cluster (< 10 elephants)	19 (0.20/0.80)
	Medium cluster (10–50 elephants)	372 (0.30/0.70)
	Large cluster (> 50 elephants)	603 (0.32/0.68)
Border contact with Myanmar	Yes	77 (0.19/0.81)
	No	917 (0.32/0.68)
Sampling period (months)	April–October	824 (0.30/0.70)
	November–March	170 (0.38/0.62)

^1^*CM* = Chiang Mai, *LP* = Lampang, *CR* = Chiangrai, *SKT* = Sukhothai, *Tak* = Tak, *CP*=Chaiyapum, *AY* = Ayuttaya, *NKPT* = Nakhonpathom, *RBR* = Ratchburi, *PJ* = Prajuobkirikhan, *CBR* = Chonburi, *Trat* = Trat, *BRR* = Burirum, *NKSM* = Nakhoratchsima, *SR* = Surinth, *SRTN* = Suratthani, *PNG* = Phang-nga, *Smui* = Smui, *SKL* = Songkla and *CPo* = Chumporn

^2^Defined as number of camps (i.e., those within a radius of 2 km) that shared resources like a river, road or land area, or working area during the day

A 5- to 7-ml blood sample was collected from an auricular vein. Samples were transferred to blood collection tubes and kept at room temperature for 1–2 h before centrifugation to harvest serum. Serum samples were stored frozen (− 20 °C) at the laboratory research unit of the NEI until analysis. One blood sample per elephant was used for this cross-sectional study, and randomly selected when multiple samples were available. Serum samples were thawed, diluted 1:100 and 1:200 in phosphate-buffered saline, and assessed for the presence of antibodies using the EEHV gB specific capture ELISA described by [23]. Results were expressed as OD ratios (OD sample/OD background) for both serum dilutions (1:100 and 1:200).

### Statistical analysis

A single serum dilution (1:100 and 1:200) was considered positive when the OD ratio was ≥ 3, undetectable at < 2, and inconclusive between 2 and 3 [23]. An animal was deemed seropositive when one or both of the serum dilutions were scored as positive. Results of univariable logistic regression analyses of the potential risk factors [elephant age, sex, camp cluster size, management type (extensive versus intensive), sampling period (wet vs. dry season) and location of camp (region) for the likelihood of an EEHV positive sample were expressed as the OR, CI and *P*-value. All potential risk factors were included in multivariable logistic regression analysis to create the model, with the exception of management type due to a high correlation with region. Region was chosen to be included in the full model because the model fit was better than the full model with management type. The AIC was used in a backward procedure to select the best model (smaller AIC is better). All analyses were performed using R version 3.3.0; 2016-5-3 [25].

## Results

### Descriptive analysis

**Fig. 1:**
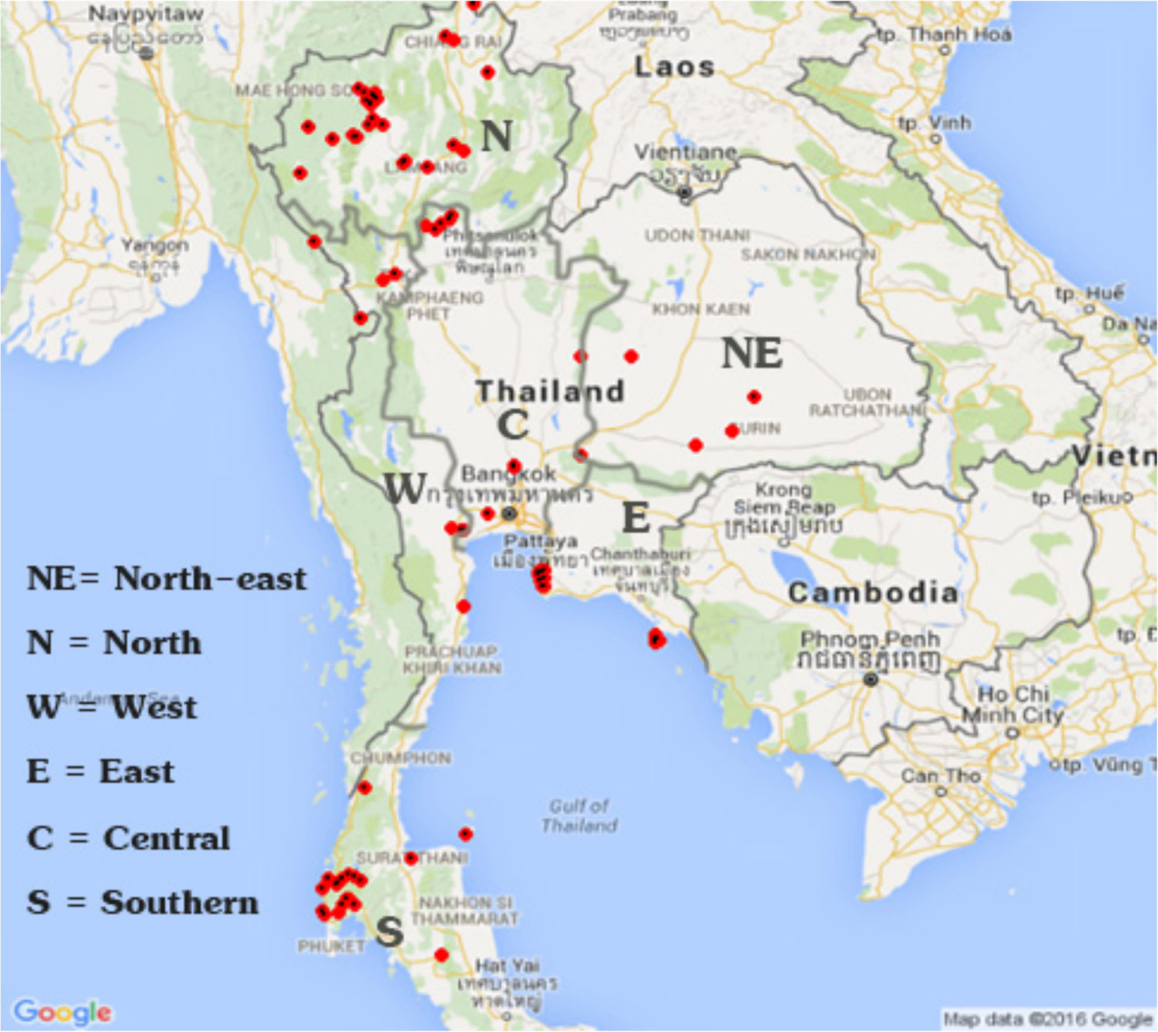
Locations of camps or clusters of camps with captive elephants (N = 994) enrolled in the present study are indicated with red dots. Red dots marked with a black dot indicate sites that had at least one EEHV antibody seropositive elephant

Animals were housed at 96 camps in 20 provinces throughout Thailand (Fig. 1); however, it was not possible to sample all elephants at every camp because elephants often were working or otherwise not available when visited by the veterinarian. We sampled one to 57 elephants per camp: at 48 camps, five or more elephants were sampled; at 25 camps, two to four elephants were sampled, and at 23 camps, only a single elephant was sampled. The interval between the first and last sampling date within each camp varied from 0 to > 500 days, although for 60 of the 96 camps (including 23 single sample locations), sampling was done within an interval of 30 days. Due to these limitations we cannot qualify any camp as (fully) EEHV-negative or EEHV-positive. Additional file 1: Figure S1 established from the present test results, using OD ratio > 3 respectively OD ratio > 4 as cut offs! gives an impression of the proportions of positive and negative animals).

Study population demographics are presented in Table 1. Amongst the 994 elephants sampled, two-thirds were female. The average age of the total population was 33.1 years (median = 34.0), with females averaging 33.8 (sd = 14.3; range, 2–76) years, and males averaging 28.9 (sd = 14.8; range, 3–66) years. The majority (80.2%) of elephants were within the adult age category (11–50 years), with 7.3% in the < 11 years and 12.5% in the > 50 years age categories. The average number of elephants sampled per camp was 10.2 (sd = 13.3; range, 1–57), and per province was 49.7 (sd = 71.6; range, 1–286). Elephants were fairly equally distributed between the two types of management systems (Table 1), with most extensive-managed camps located in the North region (86.1%). By contrast, intensive management systems were found in all regions except the North (Table 2). Most elephants resided in large, clustered areas (60.7%), as compared to small (1.9%) and medium (37.4%) clusters (Table 2). Less than 10% of the study subjects lived along the border with Myanmar in the West and North regions (Table 1). The majority of samples were collected during the wet season (April–October, 82.9%) (Table 1).

**Table 2:** Numbers of elephants sampled in intensive and extensive management systems within six geographical regions in Thailand

Region	Management System	
	Extensive^a^	Intensive^b^
Central	0	76
East	0	207
North	435	0
Northeast	2	60
South	0	82
West	68	64

^a^Elephants are managed using more traditional methods, including daily species-specific activities, and releasing elephants into the forest (by long chains or hobbles) at night to forage and interact with tame and/or wild conspecifics (U Mar, 2006)

^b^Elephants are managed individually or in small groups, are fed entirely by humans through prepared fodder, and are tethered at night (U Mar, 2006)

### Prevalence

Serology results for both serum dilutions are shown in Additional file 2: Table S1. A summary of serology results for separate analyses of 1:100 and 1:200 dilutions is shown in Table 3. Of the samples designated as positive using a 1:100 dilution, ~ 88% were also positive at 1:200. Inconclusive results were similar between the 1:100 and 1:200 dilutions (287 versus 281), respectively, whereas for the undetectable group, the 1:100 dilution identified 407 samples, of which only 285 remained undetectable at 1:200. Eighteen undetectable samples at 1:100 became positive at 1:200, as did 102 inconclusive samples. Based on the criteria that a sample was considered positive if at least one serum dilution was positive, 42.3% were seropositive (420 of 994), 57.7% were undetectable (574 of 994) and none were inconclusive (Table 3).

**Table 3:** Comparison of antibody seroprevalence based on an EEHV1A glycoprotein B protein antigen specific ELISA of elephants sampled throughout Thailand between 2010 and 2015 (*n* = 994) using serum dilutions of 1:100 and 1:200

Dilution 1:100	Dilution 1:200			
	Positive^1^	Inconclusive^2^	Undetectable^3^	Totals (1:100)
Positive^a^	263 (*)	37 (*)	0	300
	(87.7%)	(12.3%)	(0%)	
Inconclusive^b^	102 (*)	140	45	287
	(35.5%)	(48.8%)	(15.7%)	
Undetectable^c^	18 (*)	104	285	407
	(4.4%)	(25.6%)	(70%)	
Totals (1:200)	383	281	330	994

^a^Optical density (OD) ratio (OD sample/OD background) > 3

^b^OD ratio between 2 and 3

^c^OD ratio < 2

^*^At least one sample dilution was positive

### Risk factor analysis

Descriptive serology test results related to potential risk factors (Table 4) were used in the univariable logistic regression analysis (Table 5). Variables used for the modeling were elephant age, sex, camp cluster size, management type (extensive versus intensive), sampling period (wet vs. dry season) and location of camp (region). Border contact samples were unevenly distributed and applied to only two regions (North and West), so those data were not included in the univariable model. Female elephants trended towards being less often seropositive than males (OR = 1.29; *p* = 0.06), but there were no differences between the age groups. Elephants from camps utilizing extensive management systems had higher seroprevalence than those managed more intensively, with some regional differences. Compared to the North, elephants in the Central, Northeast and East regions had a lower odds for a positive sample. No association was found for the EEHV status of the sample with camp cluster size and sampling period.

**Table 4:** Antibody seroprevalence of elephants throughout Thailand (*n* = 994) between 2010 and 2015 based on an EEHV1A glycoprotein B protein antigen specific ELISA, and the proportion of samples testing positive or negative relative to potential EEHV risk factors

Potential risk factors	Positive^a^	Negative
Sex		
Female (*n* = 678)	273 (40.2%)	405 (59.7%)
Male (*n* = 316)	147 (46.5%)	169 (53.4%)
Age category		
< 11 years (*n* = 73)	32 (43.8%)	41 (56.2%)
11–50 years (*n* = 797)	331 (41.5%)	466 (58.5%)
> 50 years (*n* = 124)	57 (45.9%)	67 (54.0%)
Management type		
Extensive (*n* = 505)	238 (47.1%)	267 (52.9%)
Intensive (*n* = 489)	182 (37.2%)	307 (62.8%)
Region		
Central (*n* = 76)	17 (22.4%)	59 (77.6%)
East (*n* = 207)	78 (37.7%)	129 (62.3%)
North (*n* = 435)	215 (49.4%)	220 (50.6%)
Northeast (*n* = 62)	21 (33.9%)	41 (66.1%)
South (*n* = 82)	36 (43.9%)	46 (56.1%)
West (*n* = 132)	53 (40.2%)	79 (59.8%)
Camp cluster^b^		
< 10 (*n* = 19)	11 (57.9%)	8 (42.1%)
10–50 (*n* = 372)	164 (44%)	208 (55.9%)
> 50 (*n* = 603)	245 (40.6%)	358 (59.4%)
Border contact		
Yes (*n* = 77)	25 (32.5%)	52 (67.5%)
No (*n* = 917)	395 (43.0%)	522 (57.0%)
Evaluation period		
Apr-Oct (*n* = 824)	337 (40.9%)	487 (59%)
Nov-Mar (*n* = 179)	83 (48.8%)	87 (51.2%)

^a^Samples were considered positive if at least one dilution (1:100, 1:200) was positive (OD ratio > 3) . All other combinations were defined as negative

^b^Defined as number of camps (i.e., those within a radius of 2 km) that shared resources like a river, road or land area, or working area during the day

**Table 5:** Univariable regression analysis of potential risk factors for the presence of EEHV antibodies in elephants sampled throughout Thailand between 2010 and 2015 (*n* = 994) based on an EEHV1A glycoprotein B protein antigen specific ELISA

Potential risk factors	Prevalence (%)	*p*-Value	OR	95% CI
Sex				
Female (*n* = 678)	40.2	Ref	1	NA
Male (*n* = 316)	46.5	0.06	1.29	0.98–1.68
Age category (year)				
< 10 (*n* = 73)	43.8	Ref	1	NA
10–50 (*n* = 797)	41.5	0.70	0.91	0.56–1.48
> 50 (*n* = 124)	45.9	0.77	1.09	0.61–1.95
Management type				
Extensive (*n* = 505)	47.1	Ref	1	NA
Intensive (*n* = 489)	37.2	0.00	0.66	0.51–0.85
Region				
North (*n* = 435)	49.4	Ref	1	NA
Central (*n* = 76)	22.4	< 0.00	0.29	0.16–0.51
East (*n* = 207)	37.7	0.00	0.61	0.44–0.86
Northeast (*n* = 62)	33.9	0.02	0.52	0.29–0.90
South (*n* = 82)	43.9	0.36	0.80	0.49–1.28
West (*n* = 132)	40.2	0.06	0.68	0.46–1.01
Camp cluster^a^				
< 10 (*n* = 19)	57.9	Ref	1	NA
10–50 (*n* = 372)	44.0	0.24	0.57	0.21–1.44
> 50 (n = 603)	40.6	0.13	0.49	0.19–1.24
Evaluation period				
Apr-Oct (*n* = 824)	40.9	Ref	1	NA
Nov-Mar (*n* = 170)	48.8	0.37	1.24	0.76–2.01

*Ref* reference category, *NA* not applicable, *OR* odds ratio, *CI* confidence interval

^a^Defined as number of camps (i.e., those within a radius of 2 km) that shared resources like a river, road or land area, or working area during the day

In the multivariable logistic regression analysis, only region remained in the model after backward elimination of the variables and therefore the results are the same as from the univariable model with region (Table 5).

## Discussion

The present study was the first to conduct a large cross-sectional survey of EEHV seroprevalence among captive elephants in Thailand. Using an EEHV-1A gB protein antigen ELISA [23], over 40% of elephants tested were found to be seropositive. Although animals were healthy at the time of blood collection, a significant number appeared to have been exposed to EEHV based on antibody seroprevelance, most likely maintaining this virus within the population. Because it was not possible to sample every elephant at each camp, we could not determine if there were any 100% seropositive or seronegative camps in Thailand. However, the vast majority of seronegative elephants resided at camps with seropositive ones and so could be susceptible to infection in the future. In the study of van den Doel [23], some elephants maintained significant titers for prolonged periods, while others were intermittently seropositive. One seropositive elephant was categorized as healthy at the time of blood collection, but had presented with EEHV-like symptoms a few weeks before. This finding may indicate a prior EEHV infection, but that could not be confirmed. Results suggest that routine serological surveys may help identify prior viral exposure, which would otherwise go undetected as many exposed elephants are asymptomatic.

One of the characteristics of herpes viruses is their ability to go into latency. By certain unknown stimuli these latent viruses may be reactivated [26]. If reactivation does not occur over a long period of time, antibodies may drop to levels near to or below the detection limit of the ELISA. This makes it difficult to conclude that inconclusive or seronegative elephants are actually free of EEHV. The elephants in this study were all over 1 year of age, so maternal antibodies were not likely present to influence the outcome of the ELISA. As there is no vaccine against EEHV available, all antibody titers that were detected are assumed to be the result of previous exposure to EEHV. Each elephant with antibodies against EEHV should be considered as latently infected and a potential periodical shedder [26, 27]. Camps that consist of only seronegative animals are at risk of infection if a seropositive elephant is added to that camp; however, a false seronegative status may be the result of the absence of virus reactivation over a prolonged period, or insensitivity of the ELISA to detect a significant titer. As a consequence animals newly introduced into a camp are at risk of infection depending on the presence of even only one animal classified as EEHV infected. Model building initiated by submitting sex, age, regions, camp cluster size and sampling period (without management type) to multivariable analyses gave rise to the final multiple logistic regression model that identified “regions” as the most potent risk factor to EEHV in Thailand. More specifically, our study revealed that the Central, Northeast, East, West and South regions were lower in prevalence compared to the North. This result confirmed a higher incidence of EEHV in northern regions [28] based on sample tissue submissions and reported elephant deaths. Specifically, between 2006 and 2017, 32 clinical cases of EEHV-HD in Thailand were confirmed by PCR techniques, and of those, a third (*n* = 11) were found in the North. Overall, two thirds of EEHV antibody seropositive elephants were found in North, South and West regions of Thailand. By contrast, only two cases (2/32) occurred in the Central region, an area with only a few facilities close together, with limited exchange of animals from the outside. Understanding spatial differences in seroprevalence is complicated, however, by uncontrolled/unregistered elephant movements and transfers, particularly among facilities within those regions, and so needs further study.

The type of elephant management system was a significant risk factor to positive EEHV antibody seroprevalence in the univariable model, with 47% antibody seroprevalence in extensive systems compared to 37% in more intensive systems. The North and West regions include areas along the border with Myanmar, and contain more than half of the captive elephant population in Thailand. Although there is clinical evidence of EEHV-HD in captive elephants in Myanmar, there has been no molecular confirmation to date (Charernpan P., personal communication, National Elephant Health Service, DLD Thailand, 2017). However, given the close contact and/or transport of captive elephants between the Thai-Myanmar borders, transmission of the viral disease to elephants in the North and West of Thailand from Myanmar is possible, similar to what has been documented for foot and mouth disease viral transmission across these regions [29, 30]. Captive elephants in Myanmar are maintained in more natural habitats (extensive care system), particularly at night. Most are allowed to forage in nearby forests while on long chains, so there is potential for more interaction between wild and captive elephants in that country, whereas in Thailand, captive and wild elephants are found to cohabitate mainly in western regions.

Captive elephants in the South also had a relatively high antibody seroprevalence. In general, these were working elephants from the North and West that are taken to rest at their owner’s home in the South during the low tourist season. Conversely, elephants in the Central, East and Northeast regions live in more urban areas, closer to humans, where land and especially forest, is limited, and generally they are not exchanged between camps. The camps in intensive management systems also are less likely to transport elephants or recruit elephants from outside those regions than those in extensive systems. That may limit the degree of exposure to the virus, and agrees with our finding that most elephants living in isolated areas were seronegative. It is likely the seropositive elephants that experienced recent infection or reactivation might be related to camps with frequent or with rare viral reactivations.

Elephants sampled in this study were involved in tourism or logging, which requires tame elephants; hence, the higher numbers of adults than other age categories, and females being more prevalent than males. Trending towards significance (OR = 1.29) was a sex effect, with more males being seropositive, although the relevance of this is unknown. By contrast, elephant age, camp cluster size, and sample collection period were not significant risk factors for EEHV antibody seroprevalence. Our finding that 42.3% of captive elephants in Thailand were seropositive for EEHV antibodies suggest a high rate of viral exposure in this population. Extrapolating to the total captive population in the country (*n* = 4016 elephants), upwards of 1600 may have been exposed to the virus. The EEHV antibody seroprevalence survey showed that “region” was a significant risk factor associated with the disease incidence, particularly in the North, which is likely to be related to management or perhaps genetic relationships. Since the first diagnosed case of EEHV HD in 1999, this disease has resulted in elephant deaths, particularly calves, around the world, although it is more sporadic than epidemic in captive populations. Long et al. [3] suggested that disease severity is related to primary infection, and that around 20% of young elephants are susceptible.

Finally, a potential limitation of the EEHV-1A gB protein antigen ELISA may be that it has insufficient sensitivity to detect low antibody titers, which could lead to an underrepresentation of seropositive animals. Van den Doel et al. [23] suggested that one or both OD ratios should be ≥ 3 to indicate true seropositivity, while a cut off OD ratio ≥ 4 for both would be stricter. Hence, in addition to the analysis presented in Table 4, we examined the distributions of positive and negative test results in camps using cut off OD ratios of ≥ 3 respectively ≥ 4 for both dilutions (Additional file 1: Figure S1) and found they were similar with a peak around OD ratio 2. Both distributions showed a small elevation around OD ratio 5 (dilution 1:100) or OD ratio 6 (dilution 1:200), which might indicate distribution in a population with recent reactivation of virus or recent infection, whereas the elevation around OD ratio 2 might be the mode of an uninfected population. Obviously a more strict definition of positive animals e.g. both OD ratios ≥ 4 could lead to classifications of animals with low antibody titers as EEHV-negative. Comparison of the risk factor analysis for individual elephant data, criterion for qualification as a positive animal “one or both OD ratios ≥3” (Table 5) with risk factor analysis using the more stringent qualification criterion OD ratio ≥ 4 showed the same results (Additional file 3: Table S2). Until we are able to grow virus in culture to establish ELISA sensitivities, there is the risk of misinterpreting OD ratio results, either positive or negative, depending on what cutoff value is used.

## Conclusions

This is the first comprehensive investigation of EEHV antibody seroprevalence in an Asian range country. Our study showed that 43.8% of young elephants were antibody seropositive, similar to the older age groups, which suggests that elephants of all ages are being exposed to this potentially deadly virus. Results highlight the need for additional research to determine the immunopathogenesis of an EEHV infection, especially to elucidate why antibody seroprevalence is higher in the North, and in elephants that are more extensively managed. Genomic analyses to identify potential genetic factors associated to pathogenesis also might help explain why most elephants survive, and some do not. It is particularly important to track elephants with EEHV antibody titers over longer periods of time (longitudinally), especially if they have been suspected of prior active infection.

## Additional files

Additional file 1: **Figure S1.** Stacked bar plots with numbers of negative and positive animals per herd (ranked on herd size). Using as definitions for a positive sample: both OD ratio ≥ 3 (graph a) and OD ratio ≥ 4 (graph b). (PDF 58 kb)

Additional file 2: **Table S1.** Seroprevalence based on an EEHV1A glycoprotein B protein antigen specific ELISA of elephants sampled throughout Thailand (*n* = 994) between 2010 and 2015, and the percentage of samples testing positive1, inconclusive2 or negative3 relative to potential EEHV risk factors based on different interpretation modalities: 1:100 dilution; 1:200 dilution. (PDF 58 kb)

Additional file 3: **Table S2.** Univariable regression analysis of potential risk factors for the presence of EEHV antibodies in elephants sampled throughout Thailand between 2010 and 2015 (n = 994) based on an EEHV1A glycoprotein B protein antigen specific ELISA. Seroprevalence is based on strict cutoff: positive if both OD ratio’s > 4. (PDF 52 kb)

## Abbreviations

AIC: Akaike’s Information Criterion
CI: Confidence Interval
DLD: Department of Livestock Development
EEHV: Elephant Endotheliotropic Herpesvirus
ELISA: Enzyme-Linked Immunosorbent Assay
gB: Glycoprotein B
HD: Hemorrhagic Disease
OD: Optical Density
OR: Odd’s Ratio
PCR: Polymerase Chain Reaction
qPCR: Quantitative Polymerase Chain Reaction
