# Supplementary material for: Evidence of high EEHV antibody seroprevalence and spatial variation among captive Asian elephants (Elephas maximus) in Thailand
Source: Virol J. 2019 Mar 13;16:33. doi: 10.1186/s12985-019-1142-8 (PMC6415343; doi:10.1186/s12985-019-1142-8)

Supplement Figure 1. Stacked bar plots with numbers of negative and positive animals per camp (ordered by numbers of samples) using two definitions to qualify a sample as positive: (a) both OD ratios ≥ 3; and (b) both OD ratios ≥ 4.

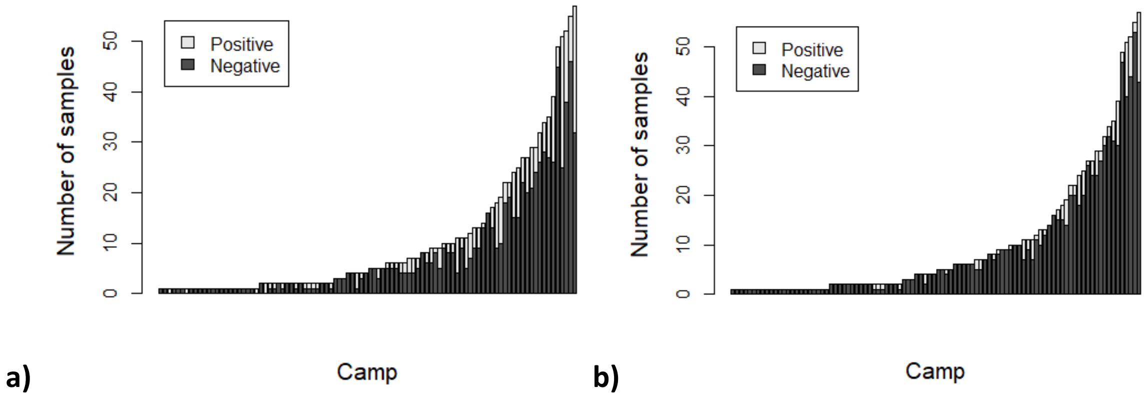

Supplement: Supplementary file 1 — Figure S1. Stacked bar plots with numbers of negative and positive animals per herd (ranked on herd size). Using as definitions for a positive sample: both OD ratio ≥ 3 (graph a) and OD ratio ≥ 4 (graph b). (PDF 58 kb) [file 12985_2019_1142_MOESM1_ESM.pdf]
