# Supplementary material for: Evidence of high EEHV antibody seroprevalence and spatial variation among captive Asian elephants (Elephas maximus) in Thailand
Source: Virol J. 2019 Mar 13;16:33. doi: 10.1186/s12985-019-1142-8 (PMC6415343; doi:10.1186/s12985-019-1142-8)
Supplement: Supplementary file 2 — Table S1. Seroprevalence based on an EEHV1A glycoprotein B protein antigen specific ELISA of elephants sampled throughout Thailand (n = 994) between 2010 and 2015, and the percentage of samples testing positive1, inconclusive2 or negative3 relative to potential EEHV risk factors based on different interpretation modalities: 1:100 dilution; 1:200 dilution. (PDF 58 kb) [file 12985_2019_1142_MOESM2_ESM.pdf]

Supplement Table 1. Seroprevalence based on an EEHV1A glycoprotein B protein antigen specific ELISA of elephants sampled throughout Thailand (n=994) between 2010-2015, and the percentage of samples testing positive[1], inconclusive[2] or negative[3] relative to potential EEHV risk factors based on different interpretation modalities: 1:100 dilution; 1:200 dilution.

Risk factors	Serum dilution 1:100			Serum dilution 1:200		
	Positive	Inconclusive	Undetectable	Positive	Inconclusive	Undetectable
Sex						
Female (n=678)	190 (28%)	200 (29.5%)	288 (42.5%)	250 (36.9%)	190 (28.0%)	238 (35.1%)
Male (n=316)	110 (34.8%)	87 (27.5%)	119 (37.7%)	133 (42.1%)	91 (28.8%)	92 (29.1%)
Age category						
<11 years (n=73)	27 (37%)	16 (21.9%)	30 (41.1%)	30 (41.1%)	20 (27.4%)	23 (31.5%)
11-50 years (n=797)	230 (28.9%)	232 (29.1%)	335 (42%)	305 (38.3%)	224 (28.1%)	268 (33.6%)
>50 years (n=124)	43 (34.7%)	39 (31.5%)	42 (33.9%)	48 (38.7%)	37 (29.8%)	39 (31.5%)

Management type						
Extensive (n=505)	165 (32.7%)	133 (26.3%)	207 (41%)	226 (44.8%)	135 (26.7%)	144 (28.5%)
Intensive (n=489)	135 (27.6%)	154 (31.5%)	200 (40.9%)	157 (32.1%)	146 (29.9%)	186 38.0%)
Region						
Central (n=76)	14 (18.4%)	27 (35.5%)	35 (46.1%)	12 (15.8%)	25 (32.9%)	39 (51.3%)
East (n=207)	58 (28%)	57 (27.5%)	92 (44.4%)	69 (33.3%)	53 (25.6%)	85 (41.1%)
North (n=435)	149 (34.3%)	118 (27.1%)	168 (38.6%)	203 (46.7%)	120 (27.6%)	112 (25.7%)
Northeast (n=62)	13 (21%)	22 (35.5%)	27 (43.5%)	17 (27.4%)	22 (35.5%)	23 (37.1%)
South (n=82)	26 (31.7%)	30 (36.6%)	26 (31.7%)	30 (36.6%)	29 (35.4%)	23 (28%)
West (n=132)	40 (30.3%)	33 (25%)	59 (44.7%)	52 (39.4%)	32 (24.2%)	48 (36.4%)
Camp cluster[1]						
<10 (n=19)	5 (26.3%)	11 (57.8%)	3 (15.7%)	11 (57.8%)	3 (15.7%)	5 (26.3%)
10-50 (n=372)	118 (31.7%)	111 (29.8%)	143 (38.4%)	148 (39.7%)	100 (26.8%)	124 (33.3%)
>50 (n=603)	177 (29.3%)	165 (27.3%)	261 (43.2%)	224 (37.1%)	178 (29.5%)	201 (33.3%)

Border contact						
Yes (n=77)	16 (20.7%)	18 (23.4%)	43 (55.8%)	25 (32.5%)	19 (24.6%)	33 (42.8%)
No (n=917)	284 (30.9%)	269 (29.3%)	364 (39.7%)	358 (39.0%)	262 (28.5%)	297 (32.4%)
Evaluation period						
Apr-Oct (n=824)	251 (30.5%)	233 (28.3%)	340 (41.3%)	313 (38.0%)	227 (27.5%)	284 (34.5%)
Nov-Mar (n=170)	49 (28.8%)	54 (31.7%)	67 (39.4%)	70 (41.2%)	55 (32.4%)	45 (26.5%)

[1] OD ratio ≥3

[2] OD ratio between 2 and 3

[3] OD ratio < 2
