# Supplementary material for: Evidence of high EEHV antibody seroprevalence and spatial variation among captive Asian elephants (Elephas maximus) in Thailand
Source: Virol J. 2019 Mar 13;16:33. doi: 10.1186/s12985-019-1142-8 (PMC6415343; doi:10.1186/s12985-019-1142-8)
Supplement: Supplementary file 3 — Table S2. Univariable regression analysis of potential risk factors for the presence of EEHV antibodies in elephants sampled throughout Thailand between 2010 and 2015 (n = 994) based on an EEHV1A glycoprotein B protein antigen specific ELISA. Seroprevalence is based on strict cutoff: positive if both OD ratio’s > 4. (PDF 52 kb) [file 12985_2019_1142_MOESM3_ESM.pdf]

Supplemental Table 2. Univariable regression analysis of potential risk factors for the presence of EEHV antibodies in elephants sampled throughout Thailand between 2010-2015 (n=994) based on an EEHV1A glycoprotein B protein antigen specific ELISA. Seroprevalence is based on strict cutoff: positive if both OD ratio's ≥ 4.

Potential risk factors	Prevalence (%)	*p*-Value	OR	95% CI
Sex				
Female (n=678)	11.1	Ref	1	NA
Male (n=316)	13.9	0.20	1.30	0.87-1.93
Age category (year)				
<10 (n=73)	13.7	Ref	1	NA
10-50 (n=797)	12.1	0.68	0.86	0.45-1.84
>50 (n=124)	10.5	0.50	0.74	0.31-1.82
Management type				
Extensive (n=505)	14.7	Ref	1	NA
Intensive (n=489)	9.2	0.01	0.59	0.40-0.87
Region				
North (n=435)	15.2	Ref	1	NA
Central (n=76)	22.4	0.02	0.23	0.06-0.64
East (n=207)	4.0	0.12	0.67	0.39-1.10
Northeast (n=62)	3.2	0.02	0.19	0.03-0.62
South (n=82)	7.3	0.07	0.44	0.17-0.98
West (n=132)	15.2	1.00	1.00	0.57-1.69
Camp cluster*				
<10 (n=19)	0.0			
10-50 (n=372)	11.3	Ref **	1	NA
>50 (n=603)	12.8	0.34	1.22	0.82-1.83
Evaluation period				
Apr-Oct (n=824)	11.4	Ref	1	NA
Nov-Mar (n=170)	19.4	0.05	1.88	0.98-3.39

Ref: reference category, NA: not applicable, OR: odds ratio, CI: confidence interval
*Defined as number of camps (i.e., those within a radius of 2 km) that shared resources like a river, road or land area, or working area during the day. ** Camp cluster <10 and 10-50 combined
